# Multifrequency bioimpedance by spectroscopy vs. routine methods in the management of hydration status in peritoneal dialysis patients: A randomized control trial

**DOI:** 10.3389/fmed.2022.911047

**Published:** 2022-09-08

**Authors:** Fabiana Lourenço Costa, Nayrana Soares do Carmo Reis, Fabricio Moreira Reis, Rogério Carvalho de Oliveira, Silméia Garcia Zanati Bazan, Maryanne Zilli Canedo da Silva, Luis Cuadrado Martin, Pasqual Barretti

**Affiliations:** Department Internal Medicine, Botucatu Medical School, São Paulo State University, Botucatu, Brazil

**Keywords:** peritoneal dialysis, overhydration, chronic kidney disease, inflammation, electrical bioimpedance, cardiovascular diseases

## Abstract

**Background:**

Overhydration (OH) is common in peritoneal dialysis (PD) and increases the cardiovascular risk. Multifrequency bioimpedance spectroscopy (BIS) has been proposed to estimate the hydration in dialysis. Our objective was to evaluate if BIS is superior than control based on clinical assessment plus single-frequency bioimpedance (SF-BIA) on the fluid control and intermediate cardiovascular outcomes.

**Methods:**

Randomized controlled study in adult PD patients, with a 9-month follow-up, allocated into two groups: control and BIS. Data were collected from medical records. SF-BIA and BIS, laboratory exams, ambulatory blood pressure monitoring, echocardiography (ECHO), and pulse wave velocity (PWV) were evaluated. The BIS data were available to the medical team only in BIS group.

**Results:**

34 patients completed the study, 17 in each group. At the endpoint the BIS group had a significant (*p* < 0.05) greater proportion of patients with OH/extracellular water (OH/ECW%) ≤ 15% than the control (94.1% vs. 52.9%), and a lower OH mean (2.1 ± 1.6 vs. 0.9 ± 1.1 L). The control group has a significant increase in the tumor necrosis factor alpha median concentration from baseline to six [11.9 (6.0–24.1) vs. 44.7 (9.4–70.6) pg/ml] and 9 months [11.9 (6.0–24.1) vs. 39.4 (27.9–62.6) pg/ml], and in the N-terminal fragment of pro-B-type natriuretic peptide median [239 (171.5–360.5) vs. 356 (219–1,555) pg/ml]. For cardiovascular parameters, BIS group presented a significant reduction in radial PWV [7.7 (6.9–9.2) vs. 6.5 (5.5–8.4) m/s] at 9 month, while in the control presented a significant increase in mean central systolic blood pressure (BP) (106.8 ± 11.2 vs. 117.6 ± 16.5 mmHg) and in central diastolic BP (90.4 ± 9.8 vs. 103.3 ± 12.5 mmHg) at 9 months. The left ventricular mass (LVM)/body surface presented a significant reduction in the control (109.6 ± 30.8 vs. 101.2 ± 28.9 g/m^2^) and BIS group (107.7 ± 24.9 vs. 96.1 ± 27.0 g/m^2^) at 9 months.

**Conclusion:**

The results suggest BIS is superior than the clinical evaluation plus SF-BIA for the fluid control of PD patients.

**Clinical trial registration:**

[https://www.ClinicalTrials.gov], identifier [RBR-10k8j3bx].

## Introduction

Overhydration, a frequent condition in end-stage kidney disease (ESKD) patients undergoing peritoneal dialysis (PD), is a major contributor to systemic arterial hypertension (SAH) (1, 2), left ventricular hypertrophy (LVH), and arterial rigidity in these patients, with a strong association with cardiovascular (CV) mortality (3, 4). In addition, increasing evidence suggests that extracellular volume overload is independently associated with a greater inflammatory response in ESKD patients (5, 6). Furthermore, overhydration *per se* can lead to CV disease in dialysis patients even in the absence of SAH (7).

Therefore, the assessment of fluid volume and its distribution among body compartments, as well as the determination of hydration status associated with blood pressure (BP) control, constitute one of the main challenges in the follow-up of dialysis patients. In routine clinical practice, the volume body water control is adjusted, in most cases, only by clinical methods, such as edema assessment, pulmonary auscultation and BP measurements, which makes it potentially inaccurate, since several clinical signs are not apparent until overhydration is advanced (8). Moreover, BP does not always reflect volume status, despite being the most used clinical tool for this assessment (9), and it is important to emphasize that to achieve a normohydration state aiming at better BP control, hypovolemia, and loss of residual renal function (RRF) may occur (10–12). This scenario reinforces the need for objective measures to determine the hydration status of these patients more accurately.

Bioelectrical impedance analysis (BIA) has been progressively gaining greater relevance in the assessment of hydration status in dialysis patients. Resistance and reactance provide information on hydration and cell integrity through the electrical properties of tissues and estimate body composition using predictive equations (10–12). There are two main BIA categories: unifrequency single-frequency bioimpedance (SF-BIA) and multifrequency (MF-BIA) (13). The main MF-BIA differential, specifically the body composition monitor (BCM) by bioimpedance spectroscopy (BIS), is the quantification of the hyperhydration (OH), which is a virtual compartment corresponding to the excess of hydration. Several studies have shown an association between OH and CV parameters, such as BP (2, 14) and left ventricular mass (LVM) (15).

The Clinical Practice Guideline for Nutrition in CKD: 2020 Update from the National Kidney Foundation’s Kidney Disease Outcomes Quality Initiative (KDOQI) (16) suggests the preferential use of the MF-BIA for body composition and volume assessment in HD patients. This recommendation cannot yet be extrapolated to PD due the lack of consistent scientific evidence. However, the guideline recommends that further research should be carried out in PD patients to determine the validity and reliability of MF-BIA measures, how to handle the data in daily practice, and how they can predict clinical outcomes.

Currently, there is a lack of studies evaluating the impact of BIA use on intermediate cardiovascular and inflammatory outcomes in PD patients. In addition, no previous study has evaluated whether the use of BIS is superior for hydration status control and intermediate outcomes compared with assessment based on clinical methods associated with SF-BIA.

Therefore, our objective was to compare the BIS as a complementary tool for hydration compared with the control based on clinical assessment associated with SF-BIA. Additionally, we aimed to evaluate the impact of BIS-based control on intermediate cardiovascular outcomes, such as BP control, arterial stiffness, blood pressure, structural and functional cardiac alterations, and inflammatory state.

## Materials and methods

We performed a randomized controlled trial involving ESKD patients undergoing PD for at least 90 days in a 9-month follow-up. The institutional Research Ethics Committee approved the study (5,411). All subjects who met the inclusion criteria and agreed to participate signed an informed consent form. We did not include patients under 18 years of age, amputees, with cardiac pacemakers, metallic implants, malignant neoplasms undergoing treatment, liver cirrhosis, active infectious diseases, unstable heart diseases, or severe left ventricular systolic dysfunction (left ventricular ejection fraction, ≤ 30%).

Patients underwent monthly evaluation, in which they registered dialytic (PD modality, ultrafilitration rate, dialysis solution glucose amount, and parameters of dialysis adequacity), nutritional, laboratory, and clinical data as use of antihypertensive, diuretic (fursemide), and other drugs, and 24-h urinary volume. For the anthropometric assessment of body volume, we used the Watson formula and adaptations (17, 18). Blood pressure (BP) was assessed with 24-h ambulatory BP monitoring (ABPM) according to the 7th Brazilian Guideline of Arterial Hypertension (19) with a Spacelabs 90202 monitor. The categorization values adopted for the definition of hypertension were systolic BP (SBP) 24 h ≥ 130 and diastolic BP (DBP) 24 h ≥ 80 mmHg (19).

### Laboratory evaluation

Routine laboratory tests included serum urea, creatinine, and albumin, 24-h urinary creatinine clearance, and blood hemoglobin concentration. Interleukin-6 (IL-6) (pg/ml), tumor necrosis factor alpha (TNF-α) (pg/ml), and NT-proBNP (pg/ml) were determined from blood venous samples that were centrifuged, aliquoted and kept frozen at –80°C until determination, using commercial kits according to the manufacturer’s instructions. The sensitivity of the ELISA kits for IL-6 and TNF-α was 4.69 pg/ml, with a detection range between 7.81 and 500 pg/ml, and the sensitivity of the ELISA kit for NT-proBNP was 0.38 ng/ml, with a detection range between 0.63 and 40 ng/ml. The ultrasensitive C-reactive protein (hsCRP) levels were determined using immunoturbidimetric method, with a sensitivity of 0.076 mg/l.

### Electrical bioimpedance bioelectrical impedance analysis

BIA assessments were performed by a skilled nutritionist with patients in the supine position and without dialysate in the abdominal cavity. For the SF-BIA assessments, we used a Biodynamics^®^ Model 450,800 μA, 50 kHz device and evaluated reactance and resistance. The equations used to obtain volume and body composition measurements were based on the proposals of Kushner and Scholler (20) and Cohn et al. (21).

For the MF-BIA assessments, we used a BIS body composition monitor (BCM, Fresenius Medical Care^®^, Bad Homburg, Germany), which measures the electrical response of 50 different types of frequencies between 5 and 1,000 kHz. BCM assumes a division of the body into 3 compartments, which are tissue mass normohydrated lean, normohydrated adipose tissue mass and OH, based on the model developed by Chamney et al. (22). Total body water (TBW), ECW, and intracellular water (ICE) were estimated using specific software provided by the manufacturer from the equation by Moissl et al. (23). OH was calculated as the difference between the measured and expected ECW in normal situations (13). Patients who presented relative OH (OH/ECW) < 15% were considered normohydrated (24).

### Echocardiography

The echocardiographic evaluations were performed by a single skilled examiner using a Vivid S6 (General Electric Medical Systems, Israel) with a multifrequency ultrasonic transducer 2.0–3.5 MHz, without prior knowledge of the patient’s group. During the procedure, patients remained in a left lateral decubitus position, with the left upper limb slightly flexed under the head. The images were obtained and analyzed following the recommendations of the American Society of Echocardiography (ECHO) (25).

Left ventricular mass (LVM) was obtained from the left ventricular posterior wall diastolic thickness, interventricular septal thickness, and left ventricular end-diastolic diameter using the formulas LVM (g) = 0.8 × {1.04 × [(IVSDT + PWDT + LVDD)3 - LVDD3]} + 0.6 [], and LVM indexed was estimated from LVM divided by body surface area (m^2^) (LVMsc) or by height (m^2.7^) (LVMh). The cutoffs for the diagnosis of left ventricular hypertrophy (LVH) were LVMsc > 95 g/m^2^ for women and > 115 g/m^2^ for men (26) or a gender-independent LVMh ≥ 51 g/m^2.7^ (27).

### Pulse wave velocity

To assess arterial stiffness, pulse wave velocity (PWV) was measured between the carotid-radial and carotid-femoral segments using the SphygmoCor CPV^®^ device (Atcor Medical). We used a pressure-sensitive transducer (TY-306), which was initially positioned over the carotid artery and later over the radial and femoral arteries. The exam reflects the speed that the wave takes to travel this path, using the electrocardiogram to synchronize with the cardiac cycle. From the waveforms collected, we calculated the Amplification Index (AIx), which is the difference between the first and second systolic peaks and expressed as a percentage of the magnitude of the reflected wave. From the software of this device, we obtained the central BP.

### Study groups

Patients were divided into two groups, namely, the control and BIS groups. The patients from both groups performed four assessments over the follow-up below:

1. Initial: clinical assessment, SF-BIA, BIS, laboratory assessment, ECHO, PWV, and 24-h ABPM.
2. Three months: clinical evaluation, SF-BIA, and BIS.
3. Six months: clinical evaluation, SF-BIA, and BIS.
4. Nine months: clinical assessment, SF-BIA, BIS, laboratory assessment, ECHO, PWV, and 24-h ABPM.

In the control group, the BIS measurements were not made available to the medical team, so these measurements were not used to guide clinical and dialysis prescriptions. In contrast, the results obtained by the BIS were made available to the medical team in the BIS group and could be used as a basis for clinical and dialysis prescriptions. Clinical interventions were freely chosen by the team in charge and could be: increasing the glucose concentration of the dialysis bags, changing the dose of diuretics, in addition to guidance on sodium and fluid intake.

### Statistical analysis

We estimated the sample size at 21 subjects for each group, which was necessary to obtain a 5% difference in the measure of relative hyperhydration (OH/AEC%) between the groups, with an estimated standard deviation of 4.8%, at a level of significance and power of 90%. This difference stems from a previous study (28) in which hyperhydrated patients had an OH/AEC% of 20.2%, that is, approximately 5% above the literature cutoff of 15% (29).

Randomization was performed in fixed-size blocks of four participants and stratified based on residual renal function (RRF) categorized as present or absent. To estimate the RRF, we used the mean urea and creatinine clearances, with a cutoff value of 4. Values <4 were considered the absence of RRF, and values ≥4 were considered RRF present. This value was based on a Spanish study (30) and agreed with the median found at the beginning of the study. The tool used can be found at https://www.sealedenvelope.com/ (Randomization and online databases for clinical trials) (31). This tool is a collaboration between some academic institutions and the National Health Services (NHS). Randomization was performed after inserting the randomization seed in the web application input field. In addition, in this tool, it was possible to choose the size of the blocks and the stratification of the groups. Two principles were subsequently followed: allocation concealment and intention-to-treat.

The results were expressed as the mean ± standard deviation, median (interquartile range) or percentage, according to the characteristics of each variable. To determine the normality of the data distribution, we used the Shapiro–Wilk test. To compare the groups (control and BIS and their evaluation moments), we used variance analysis (ANOVA) or its non-parametric equivalent (Kruskal–Wallis test), followed by the *post hoc* Bonferroni test. Temporal changes in variables within the same group were analyzed by mixed ANOVA for repeated measures or by the Friedman test, followed by the *post hoc* Bonferroni test. Student’s *t*-test, Wilcoxon test, paired *t*-test or chi square test were used in the other comparisons according to the type and distribution of each variable. For all analyses, we used the software R version 4.0.2, and the criterion of statistical significance corresponded to a *p*-value < 0.05.

## Results

**FIGURE 1:**
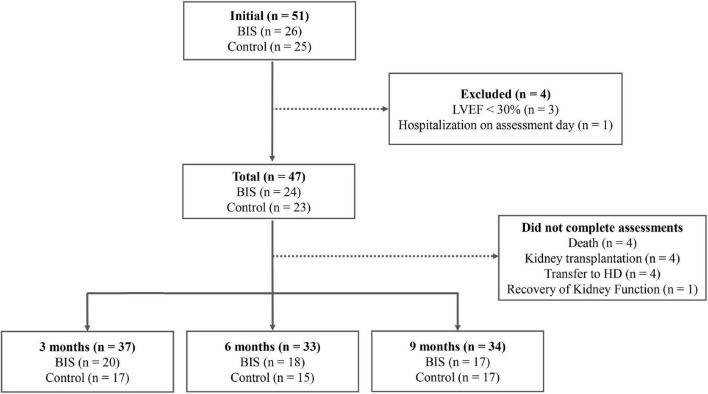
Flowchart of the participants in the different evaluation moments. LVEF, Left ventricular ejection fraction.

We enrolled a total of 51 patients allocated into the BIS (*n* = 26) and control groups (*n* = 25). Four patients were excluded after baseline assessments. Therefore, the sample consisted of 47 patients, 24 in the BIS group and 23 in the control group. During the follow-up period, four patients did not undergo the 3-month evaluation (two patients in the control group and two in the BIS group), while five patients did not perform the 6-month assessment (three patients in the control group and two in the BIS group) due to changes in the routine of outpatient medical consultations due to the COVID-19 pandemic, but they completed the 9-month follow-up. The flowchart of the subjects in the different periodic assessments is shown in Figure 1.

The demographic, clinical and dialysis characteristics of the groups at the beginning of the study are shown in Tables 1, and Table 2 shows the laboratory parameters and hydration status of the patients in both groups at the beginning of the study. There was no statistically significant difference between the control and BIS groups regarding these variables. The peritonitis rate was lower than 0.2 episodes/patient/year in both groups, with no significant difference.

**TABLE 1:** Demographic, clinical, and dialysis characteristics of the control and BIS groups at baseline.

	Total (*n* = 47)	Control (*n* = 23)	BIS (*n* = 24)	*p*
Age (years)	58.0 (44.0–66.5)	54.0 (40.5–64.5)	63.5 (50.0–69.0)	0.085
Male *n* (%)	27 (57.4)	15 (65.2)	12 (50.0)	0.292
Weight (kg)	77.7 ± 15.7	76.3 ± 18.2	78.9 ± 13.2	0.576
BMI (kg/m^2^)	28.5 ± 4.7	27.4 ± 5.0	29.5 ± 4.2	0.126
PD vintage (months)	8.9 (6.3–22.3)	8.8 (6.6–10.8)	10.7 (5.8–30.5)	0.338
DM *n* (%)	18 (38.3)	9 (39.1)	9 (37.5)	0.908
Active smoking (%)	4 (8.5)	3 (13.0)	1 (4.2)	0.276
Primary disease of ESKD n (%)				0.288
SAH	12 (25.5)	4 (17.4)	8 (33.3)	
DM	7 (14.9)	6 (26.1)	1 (4.2)	
Glomerulopathies	9 (19.1)	5 (21.7)	4 (16.7)	
Others	19 (40.5)	8 (34.8)	11 (45.8)	
PD modality (%)				0.846
CCPD	17 (36.2)	8 (34.8)	9 (37.5)	
NIPD	30 (63.8)	15 (65.2)	15 (62.5)	
Kt/V total	2.17 (1.82–2.38)	2.17 (1.96–2.31)	2.14 (1.66–2.53)	0.966
PET (%)				0.712
High	1 (2.1)	0 (0.0)	1 (4.2)	
High-average	17 (36.2)	9 (39.1)	8 (33.3)	
Low-average	26 (55.3)	13 (56.5)	13 (54.2)	
Low	3 (6.4)	1 (4.3)	2 (8.3)	
UF volume (ml/24 h)	1,050 (700–1,520)	1,200 (700–1,500)	1,005 (700–1,542)	0.898
Urine volume (ml/24 h)	1137.1 ± 716.9	1,320 ± 805.9	961.8 ± 584,2	0.087
Systolic BP (mmHg)	139.1 ± 27.3	137.0 ± 25.5	141.0 ± 29.3	0.624
Diastolic BP (mmHg)	82.7 ± 15.9	83.0 ± 15.8	82.4 ± 16.4	0.887

Values are presented as mean ± standard deviation, median, and interquartile range or percentage. *p* < 0.05.

BMI, Body mass index; PD, Peritoneal dialysis; ESKD, End-stage kidney disease; DM, Diabetes mellitus; SAH, Systemic arterial hypertension; CCPD, Continuous cycling peritoneal dialysis; NIPD, Nocturnal intermittent peritoneal dialysis; PET, Peritoneal equilibration test; UF, Ultrafiltration; BP, Blood pressure.

**TABLE 2:** Hydration status and laboratory parameters of the control and BIS groups at the beginning of the study.

	Total (*n* = 47)	Control (*n* = 23)	BIS (*n* = 24)	*p*
**SF-BIA**				
PhA (°)	5.8 ± 1.2	5.7 ± 1.2	5.9 ± 1.1	0.531
ICW (%)	51.8 ± 4.9	52.3 ± 4.7	51.3 ± 5.3	0.506
ECW (%)	48.2 ± 4.9	47.7 ± 4.7	48.7 ± 5.3	0.506
TBW (L)	39.5 ± 8.7	40.1 ± 9.7	39.0 ± 7.9	0.681
**BIS**				
OH (L)	0.9 (0.3–2.1)	1.7 (0.4–2.5)	0.7 (0.2–1.1)	0.123
OH/ECW%	5.9 ± 10.2	7.3 ± 11.2	4.6 ± 9.2	0.367
OH/ECW *n* (%)				0.638
□≤□15%	40 (85.1)	19 (82.6)	21 (87.5)	
>15%	7 (14.9)	4 (17.4)	3 (12.5)	
CCr (ml/min/1.73 m^2^)	6.0 ± 4.2	6.8 ± 5.1	5.8 ± 3.1	0.376
Hemoglobin (mg/dl)	11.8 ± 1.4	11.8 ± 1.4	11.8 ± 1.5	0.911
Serum albumin (g/dl)	3.6 ± 0.5	3.6 ± 0.5	3.6 ± 0.5	0.913
Serum urea (mg/dl)	108.4 ± 25.6	109.4 ± 30.7	107.5 ± 20.3	0.804
Serum creatinine (mg/dl)	8.4 ± 2.8	8.3 ± 2.8	8.5 ± 2.8	0.865
IL-6 (pg/ml)	16.2 (10.8–33.3)	15.7 (6.1–33.3)	16.4 (12.9–30.1)	0.598
TNF-α (pg/ml)	17.3 ± 12.1	16.1 ± 14.8	18.5 ± 9.1	0.640
hsCRP (mg/dl)	0.4 (0.1–0.8)	0.1 (0.1–0.7)	0.4 (0.1–0.8)	0.418
NT-proBNP (pg/ml)	233 (180–360.5)	239 (171.5–360.5)	211 (182–352)	0.882

Values are presented as mean ± standard deviation, median, and interquartile range or percentage. *p* < 0.05.

SF-BIA, Single-frequency bioelectrical impedance; PhA, Phase angle; ICW, Intracellular water; ECW, Extracellular water; TBW, Total body water; BIS, Bioimpedance spectroscopy; OH, Overhydration index; OH/ECW%, Overhydration normalized for extracellular water; CCr, Creatinine clearance; IL-6, Interleukin-6; TNF- α, Tumor necrosis factor-alpha; hsCRP, High sensitivity C-reactive protein; NT-proBNP, N-terminal fragment of pro-B-type natriuretic peptide.

### Evolution of intermediate outcomes

#### Hydration status by bioimpedance

**FIGURE 2:**
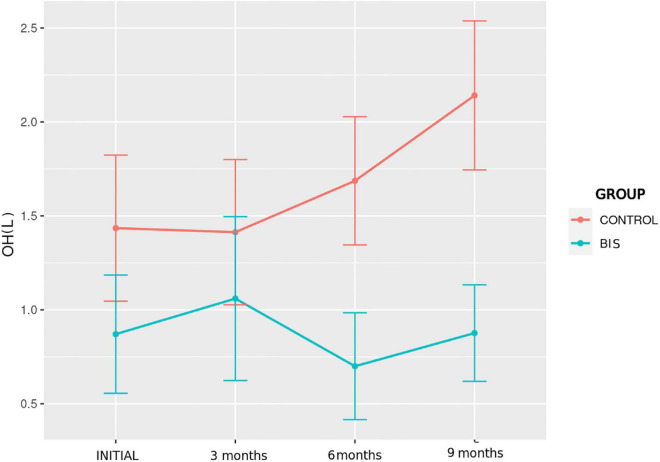
Evolution of hydration status assessed by OH over time in the study groups.

**FIGURE 3:**
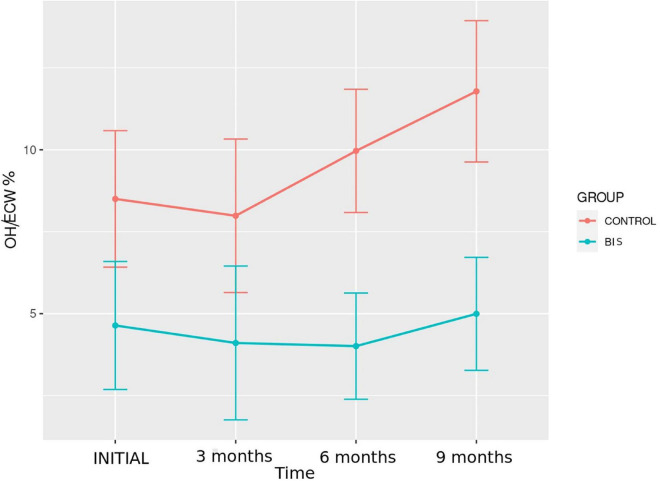
Evolution of hydration status assessed by the OH/ECW% over time in the study groups.

Data related to hydration status assessments by BIA in the control and BIS groups, performed at baseline and at3, 6, and 9 months, are shown in Table 3 and Figures 2, 3. From the comparison by mixed ANOVA, the variable OH showed a significantly different evolution over time between the groups (*p* = 0.010), with lower values observed in the BIS group (Figure 2). *Post hoc* analysis showed a significant difference between the control and BIS groups for this variable at the final moment (2.1 ± 1.6 vs. 0.9 ± 1.1 L; *p* = 0.019). The OH/AEC% showed a significantly different evolution among the groups (*p* = 0.025), with lower values observed in patients in the BIS group (Figure 3). The *post hoc* analysis showed a significant difference between participants in the control and BIS groups for this variable at the final moment (11.8 ± 8.9 vs. 5.0 ± 7.1%; *p* = 0.025).

**TABLE 3:** Evolution of hydration measurements by BIA, inflammatory markers, NT-proBNP, residual urine volume, UF volume, dialysate glucose, and diuretic dose in the evaluation moments between the different groups.

	Control				BIS			
	Initial (*n* = 23)	3 months (*n* = 17)	6 months (*n* = 15)	9 months (*n* = 17)	Initial (*n* = 24)	3 months (*n* = 20)	6 months (*n* = 18)	9 months (*n* = 17)
**SF-BIA**								
PhA (°)	5.7 ± 1.2	5.5 ± 1.3	5.5 ± 1.0	5.2 ± 1.0	5.9 ± 1.1	5.8 ± 1.2	5.8 ± 1.1	5.6 ± 1.1
ICW (%)	52.3 ± 4.7	51.8 ± 5.1	52.0 ± 3.4	50.8 ± 4.2	51.3 ± 5.3	50.3 ± 5.6	50.4 ± 5.9	50.7 ± 5.2
ECW (%)	47.7 ± 4.7	48.2 ± 5.1	48.0 ± 3.4	49.2 ± 4.2	48.7 ± 5.3	49.7 ± 5.6	49.6 ± 5.9	49.3 ± 5.2
TBW (L)	40.1 ± 9.7	39.8 ± 9.6	39.1 ± 9.0	39.2 ± 8.8	39.0 ± 7.9	39.1 ± 8.2	38.2 ± 5.4	37.4 ± 6.8
**BIS**								
OH (L)	1.7 (0.4–2.5)	1.4 ± 1.5	1.7 ± 1.3	2.1 ± 1.6**^a^**	0.7 (0.2–1.1)	1.1 ± 2.0	0.7 ± 1.2	0.9 ± 1.1**^a^**
OH/AEC%	7.3 ± 11.2	8.0 ± 9.1	10.0 ± 7.3	11.8 ± 8.9**^a^**	4.6 ± 9.2	5.1 ± 10.8	4.0 ± 6.9	5.0 ± 7.1**^a^**
IL-6 (pg/ml)	15.7 (6.1–33.3)	–	23.7 (9.4–33.9)	28.4 (18.4–36.8)	16.4 (12.9–30.1)	–	28.1 (14.0–39.2)	23.3 (16.8–32.8)
TNF-α (pg/ml)	11.9 (6.0–24.1)^b^	–	44.7 (9.4–70.6)**^b^**	39.4 (27.9–62.6)^b^	18.0 (12.9–23.6)	–	24.6 (17.1–65.9)	29.2 (12.8–32.7)
hsCRP (mg/dl)	0.1 (0.1–0.7)	–	0.2 (0.1–0.6)	0.3 (0.0–0.6)	0.4 (0.1–0.8)	–	0.6 (0.2–1.8)	0.5 (0.1–0.9)
NT-proBNP (pg/ml)	239 (171.5–360.5)**^b^**	–	287 (215–929)	356 (219–1,555)**^b^**	211 (182–352)	–	234 (205–409.5)	252 (215–1,529)
Urine volume (ml/24 h)	1,320 ± 805.9	–	1,155 ± 752.8	1,097.5 ± 873.7)	961.8 ± 584.2	–	862.2 ± 461.3	788.8 ± 495.8
UF volume (ml/24 h)	608.5 (351.2–830.2)**^b^**	–	738.5 (472.5–923)	952.5 (652.2–1,275)**^b^**	623.2 (431.9–902.1)	–	697.2 (558.4–916.7)	704.0 (500–1079.5)
Dialysate glucose (g)	200 (171.5–268.8)		225 (190.6–296.9)	250 (187.5–305)	221.4 (164.6–247)	–	206.7 (171–278.1)	200 (172.5–249)
Diuretic (furosemide) dose (mg/day)	240 (100–240)	–	240 (100–240)	240 (100–240)	240 (115–240)	–	240 (220–240)	240 (220–240)

^a^*p* < 0.05: comparison between the groups.

*^b^p* < 0.05: comparison between assessment times in the same group.

Values are presented as mean ± standard deviation, median, and interquartile range. p < 0.05. SF-BIA, Single-Frequency Bioelectrical Impedance; PhA, Phase Angle; ICW, Intracellular Water; ECW, Extracellular Water; TBW, Total Body Water; BIS, Bioimpedance Spectroscopy; OH, Overhydration Index; OH/ECW%, Overhydration normalized for extracellular water; IL-6, Interleukin-6; TNF- α, Tumor Necrosis Factor-alpha; hsCRP, High sensitivity C-reactive protein; NT-proBNP, N-terminal fragment of pro-B-type natriuretic peptide; UF, Ultrafiltration.

Comparing the participants in the control and BIS groups regarding the percentage of patients categorized in terms of hydration according to OH/AEC%, a significant difference was found at the final moment. The BIS group had a higher proportion of participants with OH/AEC% < 15% than the control group (94.1% vs. 52.9%; *p* = 0.007).

In the intragroup evaluation, by mixed two-way ANOVA with repeated measures, there were no significant differences over time regarding the hydration parameters in both groups.

#### Inflammatory markers and N-terminal fragment of pro-B-type natriuretic peptide

**FIGURE 4:**
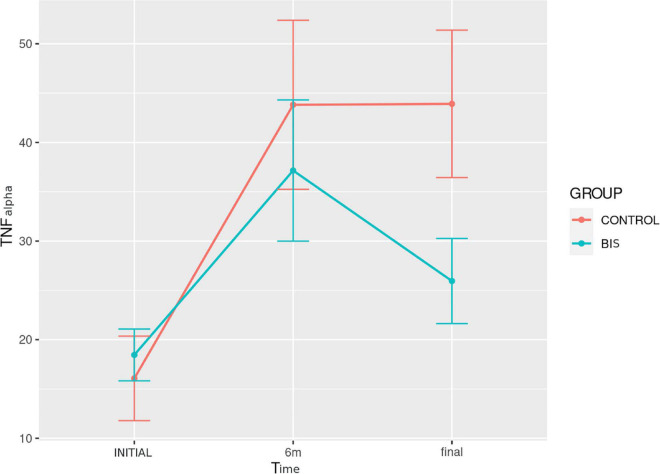
Evolution of TNF-α concentration over time in the study groups.

Table 3 and Figure 4 show the data of the measurements of IL-6, TNF-α, hsCRP, and NT-proBNP concentrations in both groups, performed at the beginning, 6 and 9 months of the follow-up. There were no differences regarding these parameters between the groups at the different evaluation moments.

**FIGURE 5:**
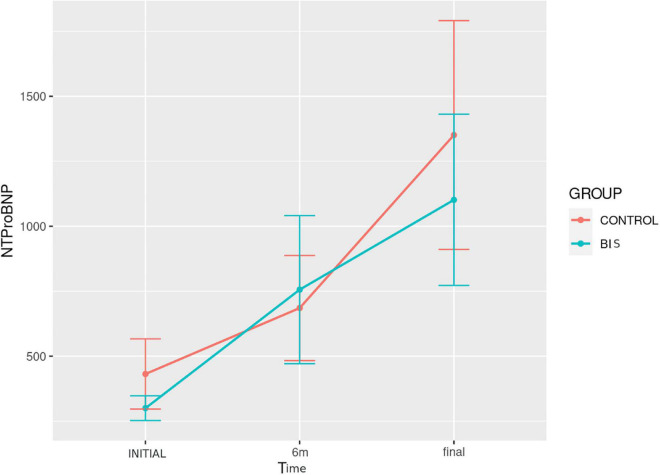
Evolution of NT-proBNP concentration over time in the study groups.

Comparing the assessment times in each study group, TNF-α had a significantly different evolution over time (*p* < 0.001) in the control group (Figure 4). In this group, *post hoc* analysis revealed a significant increase in the comparison between baseline and 6 months [11.9 (6.0–24.1) vs. 44.7 (9.4–70.6) pg/ml; *p* = 0.011], as well as between baseline and 9 months [11.9 (6.0–24.1) vs. 39.4 (27.9–62.6) pg/ml; *p* = 0.013]. For NT-proBNP concentration, there was a significantly different evolution over time (*p* = 0.001), with an increase in both groups (Figure 5). In turn, *post hoc* analysis showed a significant difference between baseline and 9 months [239 (171.5–360.5) vs. 356 (219–1,555) pg/ml; *p* = 0.027] only in the control group.

#### Cardiovascular parameters

The results of cardiovascular assessments and the use of antihypertensives at baseline and after 9 months are shown in Table 4. There was a significant reduction in radial PWV [7.7 (6.9–9.2) vs. 6.5 (5.5–8.4) m/s; *p* = 0.012] and cardiac output (5.5 ± 0.6 vs. 4.9 ± 0.6 L/min; *p* = 0.015) in the BIS group at 9 months in comparison with baseline.

**TABLE 4:** Evolution of cardiovascular parameters and antihypertensive medication use at baseline and at 9 months in the study groups.

	Control			BIS		
	Initial (*n* = 23)	9 months (*n* = 17)	*p*	Initial (*n* = 24)	9 months (*n* = 17)	*p*
Radial PWV (m/s)	7.7 (6.8–8.6)	6.9 (5.8–7.2)	0.084	7.7 (6.9–9.2)	6.5 (5.5–8.4)	0.012
Femoral PWV (m/s)	9.2 (7.5–11.9)	8.7 (7.6–9.8)	0.394	10.2 (7.5–13.1)	8.1 (6.9–12.1)	0.842
SBP 24 h (mmHg)	124.5 (114.0–141.3)	137.5 (123.8–143.5)	0.084	124.0 (107.0–139.0)	124.0 (119.0–135.0)	0.480
DBP 24 h (mmHg)	78.7 ± 10.3	82.2 ± 11.9	0.227	74.3 ± 13.0	76.7 ± 10.1	0.385
Mean Central SBP (mmHg)	106.8 ± 11.2	117.6 ± 16.5	0.009	110.0 ± 16.6	114.9 ± 19.0	0.257
Central SBP (mmHg)	124.6 ± 20.8	124.3 ± 14.5	0.634	123.3 ± 20.2	116.7 ± 17.6	0.773
Central DBP (mmHg)	90.4 ± 9.8	103.3 ± 12.5	0.003	93.5 ± 14.8	97.1 ± 15.0	0.466
LVM (g)	204.5 ± 64.4	190.4 ± 69.7	0.028	203.5 ± 51.3	177.9 ± 48.6	0.053
LVMh (g/m^2,7^)	52.0 ± 15.0	48.0 ± 16.0	0.011	53.8 ± 13.1	46.9 ± 15.7	0.074
LVMsc (g/m^2^)	109.6 ± 30.8	101.2 ± 28.9	0.010	107.7 ± 24.9	96.1 ± 27.0	0.030
LAVsc (ml/m^2^)	29.9 (24.5–35.2)	28.5 (24.1–33.9)	0.142	33.7 (28.1–38.6)	27.9 (24.5–31.8)	0.182
LVDD (mm)	48.0 (46.0–49.5)	47.0 (45.0–50.0)	0.057	48.0 (47.0–49.0)	47.0 (45.5–51.3)	0.227
LVSD (mm)	29.0 (27.0–31.0)	29.0 (26.0–31.0)	0.089	29.0 (28.0–31.5)	29.0 (27.0–34.0)	0.843
CO (L/min)	5.2 ± 0.6	5.2 ± 1.1	0.926	5.5 ± 0.6	4.9 ± 0.6	0.015
LVEF (%)	65.4 ± 10.5	63.2 ± 12.9	0.300	64.6 ± 8.2	65.1 ± 6.3	0.642
Number of antihypertensive classes	2.0 (2.0–3.0)	2.0 (2.0–3.0)	0.152	2.5 (2.0–3.0)	3.0 (2.0–3.0)	0.491
SAH *n* (%) (SBP 24 h ≥ 130 mmHg)	8 (36.4)	7 (58.3)	0.383	9 (39.1)	4 (30.8)	0.888
**LVH *n* (%)**						
LVMh > 51 g/m^2,7^	13 (56.5)	9 (41.2)	0.923	12 (52.2)	4 (26.7)	0.223
LVMsc > 95 g/m^2^ W/ > 115 g/m^2^ M	12 (52.2)	7 (41.2)	0.713	13 (56.5)	4 (26.7)	0.140

Values are presented as mean ± standard deviation, median, and interquartile range or percentage. *p* < 0.05.

PWV radial, Pulse wave velocity carotid-radial; PWV femoral, Pulse wave velocity carotid-femoral; SBP, Systolic blood pressure; DBP, Diastolic blood pressure; LVM, Left ventricular mass; LVMh, Left ventricular mass indexed to height; LVMsc, Left ventricular mass indexed to body surface area; LAVsc, Left atrial volume indexed to body surface area; LVDD, Left ventricular diastolic diameter; LVSD, Left ventricular systolic diameter; CO, Cardiac output; LVEF, Left ventricular ejection fraction; LVH, Left ventricular hypertrophy; W, Women; M, Men.

Mean central SBP increased significantly in the control group (106.8 ± 11.2 vs. 117.6 ± 16.5 mmHg; *p* = 0.009), as did central DBP (90.4 ± 9.8 vs. 103, 3 ± 12.5 mmHg; *p* = 0.003) at the ninth month.

There was a significant reduction in LVM (204.5 ± 64.4 vs. 190.4 ± 69.7 g; *p* = 0.028) and LVMh (52.0 ± 15.0 vs. 48.0 ± 16.0 g/m; *p* = 0.011) in participants in the control group. In turn, LVM sc was significantly reduced in both the control group (109.6 ± 30.8 vs. 101.2 ± 28.9 g/m^2^; *p* = 0.010) and the BIS group (107.7 ± 24.9 vs. 96, 1 ± 27.0 g/m^2^; *p* = 0.030). There was no significant difference regarding the other variables.

#### Residual diuresis, ultrafiltration volume, dialysate glucose amount, and use of diuretics

Table 3 presents data on 24-h urine volume, UF volume (data from the patients’ personal notes at consultations), dialysate glucose amount, and diuretic dose in both groups, performed at baseline, 6 and 9 months of follow-up. There were no differences regarding these parameters between the groups at the different evaluation times.

Comparing the assessment times in each study group, the UF volume had a significantly different evolution over time (*p* = 0.02) in the control group. In this group, *post hoc* analysis revealed a significant increase in the comparison between baseline and 9 months [608.5 (351.2–830.2) vs. 952.5 (652.2–1,275) ml; *p* = 0.04]. There were no significant differences in the percentage of patients using furosemide and its dose over time, as well as in 24-h urine volume, RRF, and dialysate glucose.

## Discussion

Our results showed that the use of BIS, using a BCM, had a positive impact on hydration control in PD patients, corroborating the results of Luo et al. (32). We observed lower OH and OH/AEC at the final follow-up in patients in the BIS group. In addition, the frequency of patients with OH/AEC, 15%, was greater in patients in the BIS group than in participants in the control group at the ninth month, suggesting better fluid control. This finding has great relevance since, in previous studies, even in HD patients, the volume overload assessed by this method is a strong predictor of cardiovascular mortality (33, 34). Kim et al. (35) also reported that exposure to hyperhydration over 1 year was able to predict a shift to HD and the risk of death in PD patients.

Regarding NT-proBNP, our study showed a significant increase in its levels only in the control group, in agreement with the findings obtained by BIS. Park et al. (36), in a cross-sectional study, evaluated body composition in HD and PD patients using BCM and NT-proBNP concentrations for hydration analysis. These authors found significant associations between OH/AEC and higher NT-proBNP levels. Likewise, Wang et al. (37) evaluated 129 HD patients and reported an association between NT-proBNP levels and hyperhydration assessed by BCM, concluding that the BIS is a reliable marker of volume status.

Extracellular volume expansion is possibly associated with the inflammatory response in CKD patients, as suggested by several authors (5, 6, 38). Volume overload could elicit inflammation by the translocation of endotoxins from the edematous intestinal loops or by the sodium direct tissue effect (39). Previous results from our group showed that dietary sodium restriction (5) as well as reduced sodium concentration in hemodialysis dialysis solution (40) were significantly associated with reduced IL-6 and TNF-α concentrations. In this study, the TNF-α concentration significantly increased throughout the follow-up period, only in the control group, which had more parameters of hyperhydration.

Chronic fluid overload also contributes to increased arterial stiffness, which occurs due to structural and functional changes in the vascular wall, a recognized risk factor for the development of cardiovascular diseases (18, 41). The most validated method for arterial stiffness evaluation is carotid-femoral PWV (42). In the present study, there was no significant difference between the groups regarding these parameters. However, there was a reduction in carotid-radial PWV in the BIS group at 9 months compared with baseline, which suggests a decrease in arterial stiffness.

In agreement with our results, Kocyigit et al. (43) observed a higher PWV in the hyperhydrated compared to normohydrated patients in PD patients stratified according to OH. They also found a positive correlation between NT-proBNP concentration and OH/AEC with PWV, and OH/AEC was an independent predictor of PWV. The authors suggested that reducing hyperhydration would potentially reduce arterial stiffness.

Left ventricular hypertrophy is quite prevalent in PD, since ESKD patients have a high prevalence of arterial hypertension, anemia, and volume overload, which is a well-established risk factor for the development of eccentric LVH (44). In this study, there was a reduction in LVMsc in both study groups at the final follow-up. However, LVM and LVMh were reduced only in the control group and at the ninth month of the follow-up. It is important to interpret this result with caution, even though some authors have proposed LVMh as a more adequate parameter for dialysis patients (27).

Even though the 24-h ABPM measurements were not different between the groups, there was a significant increase in mean central BP, measured by the PWV, in the control group. Although they are more costly and non-routine, this BP approach allows for a broader investigation of the cardiovascular system, being more relevant than peripheral measures regarding the pathogenesis of cardiovascular diseases (18, 45, 46).

Our results on ultrafiltration rate, diuretic dose, dialysate glucose amount showed higher glucose use (without reaching statistical significance) and significant higher UF rate in the control group (Table 3). This apparently paradoxical result may reflect greater difficulty of hydration control in the control group, which despite greater UF without reduction of urinary volume presented markers of hyperhydration. Although it is not possible to recover data on water intake and reliable sodium intake, we can speculate that patients in the BIS group received more guidance regarding the control of sodium and water intake.

This study has several limitations, particularly the small number of subjects who completed the 9-month follow-up, which reduces its statistical power. In addition, we were not able to quantify the water and sodium intake over the study, a data that could explain our findings. Also, it was not possible to use inferior vena cava diameter assessment to state as outcome between the two groups. Finally, the use of drugs that interfere with the inflammatory status, such as statins, was not analyzed because most patients were using such medication during the study and had no change in dose.

Its strengths are related to the measurements by a single trained evaluator, especially ECHO. In addition, the evaluations took place at the same time, minimizing changes that could occur in the time interval between them. To our knowledge, this was the first study that included SF-BIA as a routine assessment associated with clinical methods, with the aim of evaluating whether BIS would offer additional advantages of fluid control as well as intermediate cardiovascular outcomes, including inflammation.

## Conclusion

In conclusion, our results suggest that BIS is a reliable auxiliary method for fluid control in PD patients and is superior to routine assessments, even when associated with SF-BIA, with a positive impact on fluid control and intermediate cardiovascular outcomes such as arterial stiffness and inflammatory markers. Thus, BIS can be recommended as an auxiliary tool for hydration status evaluation in PD. However, studies with a larger number of patients are necessary to confirm our results.

## Data availability statement

The datasets presented in this study can be found in online repositories. The names of the repository/repositories and accession number(s) can be found below: https://repositorio.unesp.br/handle/11449/215123.

## Ethics statement

The studies involving human participants were reviewed and approved by the Research Ethics Committee of Botucatu Medical School, Unesp. Brazil. The patients/participants provided their written informed consent to participate in this study.

## Author contributions

FC, NR, and PB were responsible for defining the study and methodology and wrote the manuscript. FC, FR, and SB performed data collection. FC, RO, PB, and LM performed data analysis and interpretation. All authors provide intellectual content to the work and approval of the final version to be published.
